# Enhancing Coccinella Beetle Biological Pest Control via a Floral Approach in Cucumber Greenhouse

**DOI:** 10.3390/life13102080

**Published:** 2023-10-19

**Authors:** Moazam Hyder, Yuyan Li, Muhammad Fahad Raza, Maosen Zhang, Junjie Chen, Jianjun Mao, Aslam Bukero, Lisheng Zhang

**Affiliations:** 1 State Key Laboratory for Biology of Plant Diseases and Insect Pests, Key Laboratory of Natural Enemy Insects, Ministry of Agriculture and Rural Affairs, Institute of Plant Protection, Chinese Academy of Agricultural Sciences, Beijing 100193, China; 2 Department of Entomology, University of Agriculture Faisalabad, Faisalabad 38000, Pakistan; fbiuaf91@gmail.com; 3 Department of Entomology, Sindh Agriculture University Tandojam, Tandojam 70050, Pakistan; abukero@sau.edu.pk

**Keywords:** aphid, whitefly, biocontrol agent efficacy, *Coccinella septempunctata*, *Menochilus sexmaculatus*, floral resources, fecundity rate

## Abstract

Flower-rich habitats are crucial for promoting biodiversity and ecosystem services within agricultural ecosystems, such as pollination and pest control. The present study investigates the efficacy of employing floral structures as a criterion for the selection of plant species in order to enhance the attraction of natural enemies within cucumber greenhouses, consequently augmenting floral resources. The results of our study provide evidence that flower strips have a beneficial effect on the fitness of critical natural predators, while not facilitating the proliferation of detrimental insect species. These findings exhibit potential for enhancing pest-management services in the agricultural sector. The findings of our study demonstrate that pest levels within greenhouse environments closely resemble those observed in real-world commercial cropping systems. As a result, the introduction of *Coccinella septempunctata* and *Menochilus sexmaculatus* biocontrol agents is confirmed to be a reliable and efficient method for pest management. The phenomenon of predator–prey density dependency is recognized as a crucial element in the implementation of biological control strategies. Furthermore, we investigate the impact of floral resources on the reproductive capacity of indigenous predators. The impact of *Coriandrum sativum* on fertility is substantial, indicating that the presence of a varied plant assortment with overlapping flowering periods can prolong the availability of floral resources. This study highlights the significance of flower-rich habitats and deliberate plant selection in augmenting biodiversity, ecosystem services, and pest management within agricultural settings. The implementation of conservation biological control technologies presents supplementary ecological advantages, thus offering practical implications for the promotion of sustainable agricultural practices.

## 1. Introduction

In the future, agriculture must balance feeding growing populations with reducing environmental impacts to achieve biodiversity conservation and food security [1]. Conventional farming practices, like mechanization and agrochemical use, have negative impacts on ecosystem processes, potentially undermining agricultural production [2,3]. Cucumbers, one of the most important cucurbitaceous vegetables, are grown in greenhouses and open fields under many different environmental conditions for both local consumption and exportation. The microclimate in greenhouses attracts sucking pests due to warm, humid conditions. *Cucumis sativus* is regarded as the fourth most important vegetable in the world, maybe because of its excellent nutritional, therapeutic, and commercial potential [4,5].

Globally, the use of biological pest management is encouraged for greenhouse crops for greenhouse plants including tomatoes, sweet peppers, and cucumbers. The lady bird beetle (*Coccinella septempunctata*) and Zigzag beetle (*Menochilus sexmaculatus*) have been employed to manage a variety of pests, such as aphids, thrips, whiteflies, mites, and lepidopteron eggs [6,7]. These insects, including aphids, thrips, whiteflies, coccicids, and psyllids, are significantly preyed upon by these species. Aphid-feeding species can be found in Pakistan, India, Borneo, Jawa Indonesia, the Philippines, the Bali Islands, France, Sumatra, and South Africa [8,9]. In conservation biological control, natural enemies are made stronger by altering their habitat [10].

Natural enemy-based biological pest management is becoming a crucial aspect of sustainable crop production [11]. Enhancing habitat variety by placing seminatural vegetation in or near agricultural fields is one way to increase biological control using localised natural enemies [12,13,14]. The most obvious potential drawback of these on-farm diversification schemes is the need to remove some land from production, which might negate any financial benefits from diversity [15]. Furthermore, some of the advantages of habitat diversification may not become apparent for a while after adoption [14,16]. These disadvantages could deter farmers from using this strategy, especially for high-value crops. The discharge of mass-reared natural enemies in huge numbers to achieve the immediate control of pests is an alternate and possibly complementary approach for enhancing biological control. In fact, augmentative releases of natural enemies in a variety of agricultural systems have been shown to be an effective and affordable substitute for chemical pest management [17].

Pollination and biodiversity preservation are two environmental services that can be supported by the use of this sort of strategy [18]. In order to boost the population of beneficial insects, floral resources can be provided within or near crops [19,20,21]. This is known as a conservation biological management strategy. Several predators and parasitoids have been shown to benefit from flowering plants in terms of growth, survival, development, and reproduction [12,22]. Energy is provided by nectar, while protein is provided by pollen. In their mature stages, aphidophagous predators such as lacewings, syrphidae, and ladybirds benefit from additional food sources [23]. When blooming companion plants are grown alongside a primary crop of sweet pepper, studies have indicated a significant increase in syrphidae abundance [24]. For the females who are growing consecutive batches of eggs, flowering plants offer food supplies, alternate prey, protection from pesticides, or a site for sex meetings. In addition, these adult syrphidae have been identified as possible strawberry crop pollinators [25,26]. For hoverflies to develop and survive in agricultural environments, the quantity and quality of these food sources are essential [27]. In order to maintain natural enemy populations for improved pest control and pollination services, more food and floral resources are required. The most prevalent varieties of flower that draw hoverflies are coriander, sweet alyssum (*Lobularia maritima* L. Desv.), *Phacelia tanacetifolia* Benth, and buckwheat (*Fagopyrum esculentum*) [28,29]. In this study, we evaluated the biocontrol potential of *Coccinella septempunctata* and *Menochilus sexmaculatus* that were employed alongside *Coriandrum sativum* plants and supplied with flower nectar inside playhouse structures that housed cucumber greenhouses. Reduced plant damage and improved pest control are benefits of augmentative biocontrol. We released predators and added floral plants to a cucumber greenhouse to test the efficacy of augmentative biocontrol. Our research sought to ascertain if this method enhances pest management while minimizing plant harm.

## 2. Materials and Methods

### 2.1. Study Location

The research was carried out in commercial cucumber greenhouses operated by Fast Grow Agri. Farm (25°30′23″ N, 68°46′22″ E), Tando Allahyar, Sindh, Pakistan. Cucumber was planted in this location in December–January and grew until July–August.

### 2.2. Insect Rearing, Release, and Sampling

The predators *M. sexmaculatus* and *C. septempunctata* were reared on fresh Akk aphids (collected from field) and an artificial diet (Gurr 2 g, Yeast 6 g, and 10 mL of water) in the Biological Control Laboratory, Department of Entomology, Sindh Agriculture University, Tandojam, Pakistan. They were maintained in wooden net cages (56 × 40 × 56 cm) under laboratory conditions of 25 ± 1 °C, 50–60% relative humidity, and a 16:8 L: D photoperiod.

*M. sexmaculatus* and *C. septempunctata* were released in the first application (1st week: 30 adults + 30 3rd instar larvae), in the second application (3rd week: 60 adults + 60 3rd instar larvae), and in the third application (5th week: 90 adults + 90 3rd instar larvae). All three release applications were applied in parallel in floral plots in the presence of *Coriandrum sativum* and without it, but not for the control. *B. tabaci* and *M. persicae* population densities were assessed in all experimental plots from 3 days after treatment (DAT) to 6 weeks. A random selection of five leaves from each plant and each row of 10 plants, representing the three plant levels (upper, middle, and bottom), was performed. The number of eggs from both coccinellid species was also counted each week, beginning in the first week and continuing through the sixth week.

### 2.3. Greenhouse

The polyhouse structure used for this experiment had two doors for natural ventilation and was made of galvanized iron pipes. It was coated with nets with a 40-mesh size and anarc-shaped domes that were UV-stabilized. The treatments were reproduced in 2 blocks and the experiment was run using a randomized complete block design (RCBD). In the experiment, two greenhouses were used; the total area of each greenhouse was 480 m square (m^2^). One was divided into ten plots (10 × 4 m^2^ each), separated by a white net to prevent the free movement of insects. Each treatment was replicated on three plots. The five treatments consisted of *Menochilus sexmaculatus* + *Coriandrum sativum* (MScR), *Menochilus sexmaculatus* (MS), *Coccinella septempunctata* + *Coriandrum sativum* (CScR), *Coccinella septempunctata* (CS), and a controlled pilot (C) in which there was no release of natural enemies. Five rows were hoed into existence in each greenhouse, providing enough space for 200 cucumber plants to be planted. Cucumber (Yaela variety Yuksel Company, Ankara, Turkey) seedlings were grown in germination trays before being transplanted in the last week of January 2021 at a row-to-row and plant-to-plant spacing of 60 cm × 30 cm. By following the recommended cultural and management practices for the production of cucumber under naturally ventilated playhouses, the crop was grown in an environment free of pesticides. A technique using bamboo pegs and nylon ropes was used to encourage the best possible development of cucumber plants. This method intended to direct cucumber plants along distinct main stems or branches, ensuring that each plant had a single, well-defined development route. To avoid overpopulation and encourage healthy growth, the extra branches were frequently pruned. Coriander seeds were also planted in greenhouses in parallel, with three pots between each row of plants. Each pot was 20 cm in diameter. With the use of this gardening approach, numerous plant species began flowering in February and peaked in March.

### 2.4. Data Analysis

Statistical analyses were conducted using the Mann–Whitney test to measure the average number of aphids and whiteflies per 10 plant samples per row, and to examine the effects of *M. sexmaculatus* and *C. septempunctata* in the presence of floral plants and without them in each plot of the greenhouse. The reduction percentage of aphids and whiteflies was determined using Abbott’s formula (Equation (1)) [30]:

(1) Reduction %= ((1−n) in T after treatment)/(n in Co after treatment)×100

where *n* is the insect population, *T* is “treated”, and *Co* is the control.

The fecundity count of natural enemies in the presence of a floral plot and without a floral plot was analysed using the means of a selected 10 plants/row, from the first week to the sixth week. Using the Tukey’s *HSD* test with a 5% significance level, the mean differences between the various treatments were examined.

## 3. Results

### 3.1. Aphid Performance in the Greenhouse

**Figure 1:**
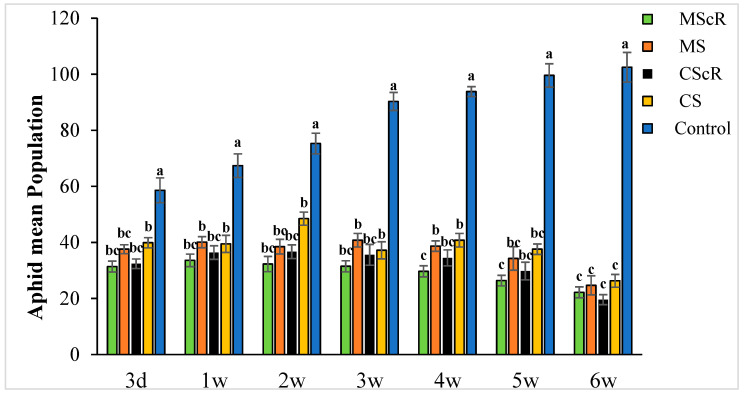
The average number (±SE) per plant of aphids in a cucumber greenhouse at different time intervals: The green bar represents MScR, light brown represents MS, black shows CScR, yellow indicates CS, and blue represents control. The mean population of *Myzus persicae* in cucumber greenhouses with the presence of natural enemies and floral plants (*Coriandrum sativum*) and natural enemies without floral plants MScR = *M. sexmaculatus* + *C. sativum*; MS = *M. sexmaculatus*; CScR = *C. septempunctata* + *C. sativum*; CS = *C. septempunctata* at different D = days and W = weeks of observation. Different letters indicate statistically significant differences between treatments. ANOVAs with Tukey HSD post hoc tests were used to compare treatments.

**Figure 2:**
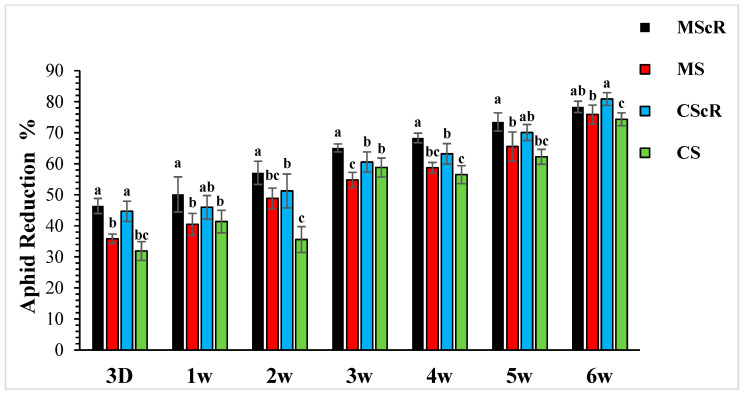
Population reduction in aphids in cucumber greenhouses at different time intervals: the aphid population decreased significantly with the passage of time. The black bar represents MScR, red represents MS, the light blue colour shows CScR, and the green colour represents the reduction in *Myzus persicae* in cucumber greenhouses with the presence of natural enemies and floral plants (*Coriandrum sativum*) and natural enemies without floral plants MScR = *M. sexmaculatus* + *C. sativum*; MS = *M. sexmaculatus*; CScR = *C. septempunctata* + *C. sativum*; CS = *C. septempunctata* at different D = days and W = weeks of observation. The reduction percentage in aphid and whitefly was determined using Abbott’s formula. Different letters indicate statistically significant differences between treatments. ANOVAs with Tukey HSD post hoc tests were used to compare treatments.

The effectiveness of *M. sexmaculatus* and *C. septempunctata* in lowering aphid populations on cucumber plants was the subject of the investigation. The results showed that interventions including these natural predators significantly reduced aphid populations, especially when paired with floral-resource plants. Both *M. sexmaculatus* and *C. septempunctata* showed considerable efficacy in reducing aphid populations on cucumber plants, Furthermore, a ANOVA analysis (as shown in Table 1) demonstrated a significant difference in the deterrent effect of the tested between all treatment and control (F = 6.03, df = 4, *p* < 0.039), as compared to the control treatment. The combination of natural predators and the availability of floral resources had a notable impact on the population sizes of aphids, as illustrated in Figure 1. Significant variations were noted in the mean number of aphids per plant when comparing different treatments with the untreated control. The populations of *M. persicae* exhibited a significant decrease in both the treatments including natural enemies and natural enemies combined with floral-resource plants in comparison to the untreated control. In the untreated control group, the number of aphids per plant was much greater, measuring 67.5 ± 4.5 one week after the application of treatments. A significant positive correlation was observed between the age of the plant and the number of aphids. The aphid populations exhibited a low initial prevalence throughout the early season; however, they subsequently experienced an increase as the plants reached maturity. This can be attributed to the presence of plant sap in the leaves, which remains available even during the flowering stage. Significantly, populations of *M. persicae* exhibited improvement in all treatments except for the MScR treatment, wherein a decline in aphid populations was detected subsequent to the second release during the third week. The application of *M. sexmaculatus* and *C. septempunctata* for release in the fifth week, following a gradual decline in aphid populations in the CScR plot, demonstrated the highest efficacy, leading to the lowest aphid count of 19.6 ± 1.8 compared to the control group, which recorded 102.5 ± 5.3 aphids per plant in the sixth week. Additionally, a distinct experiment involving the utilization of Coriander floral resources and *C. septempunctata* revealed a notable decrease of 44% in the population of *M. persicae*. The running of *C. septempunctata* at 3 DAT resulted in a minimum reduction of 31% in the population of *M. persicae*, as shown in Figure 2. The excessive abundance of aphids resulted in a marginal increase in the declining pattern observed over a period of time. The successful integration of *M. sexmaculatus* and *C. septempunctata* resulted in a significant decrease in aphid populations on cucumber plants. The time of the discharge of these beneficial insects played a pivotal role in attaining the most favourable outcomes. The population of aphids was shown to be influenced by the age of the plants, exhibiting an increase as the plants progressed towards maturity. This highlights the critical relevance of integrated pest-management tactics in effectively managing aphid populations. The population density of aphids in greenhouse agriculture is substantially impacted by the presence of natural enemies and the accessibility of floral supplies.

### 3.2. Whitefly Performance in the Greenhouse

**Figure 3:**
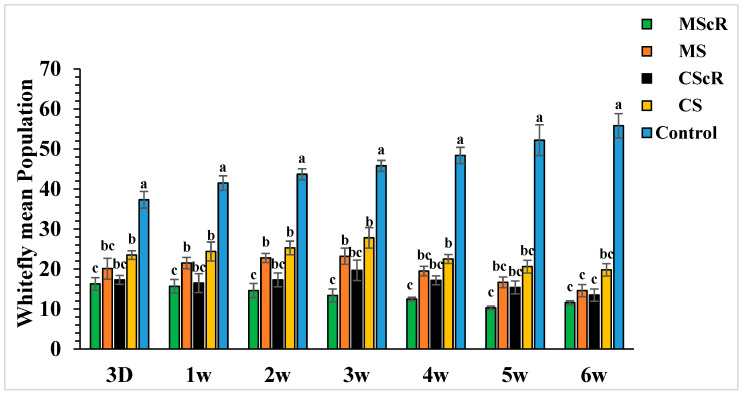
The average number (±SE) per plant of whitefly in a cucumber greenhouse at different time intervals: The green bar represents MScR, light brown represents MS, black shows CScR, yellow indicates CS, and blue represents control. The mean population of *Bamisia tabaci* in cucumber greenhouses with the presence of natural enemies and floral plants (*Coriandrum sativum*) and natural enemies without floral plants MScR = *M. sexmaculatus* + *C. sativum*; MS = *M. sexmaculatus*; CScR = *C. septempunctata* + *C. sativum*; CS = *C. septempunctata* at different D = days and W = weeks of observation. Different letters indicate statistically significant differences between treatments. ANOVAs with Tukey HSD post hoc tests were used to compare treatments.

**Figure 4:**
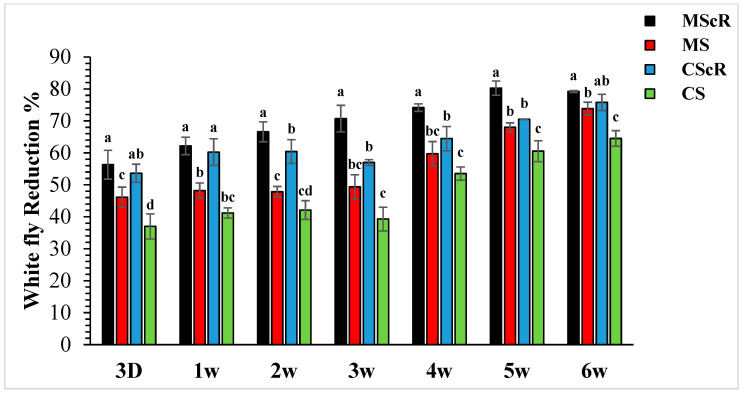
Population reduction in whiteflies in cucumber greenhouses at different time intervals: the aphid population decreased significantly with the passage of time. The black bar represents MScR, red represents MS, the light blue colour shows CScR, and the green colour represents CS. The reduction in *Bemisia tabaci* in the cucumber greenhouse with the presence of natural enemies and floral plants (*Coriandrum sativum*) and natural enemies without floral plants MScR = *M. sexmaculatus* + *C. sativum*; MS = *M. sexmaculatus*; CScR = *C. septempunctata* + *C. sativum*; CS = *C. septempunctata* at different D = days and W = weeks of observation. The reduction percentage in aphid and whitefly was determined using Abbott’s formula. Different letters indicate statistically significant differences between treatments. ANOVAs with Tukey HSD post hoc tests were used to compare treatments.

The field release performance of adults of *M. sexmaculatus* and *C. septempunctata* was evaluated in a cucumber greenhouse over a six-week infestation period (Figure 3). Both treatments continuously showed a detrimental effect on whitefly populations throughout the duration of the study, spanning from the first to the sixth week. In the third week, subsequent to the second introduction of *M. sexmaculatus* and *C. septempunctata* in all experimental areas, including the flowering plant *C. sativum*, we conducted a quantitative assessment of the population of *B. tabaci* per individual plant using statistical analysis (F = 7.70, df = 4, *p* < 0.241), as compared to the control treatment. The treatment that showed the highest efficacy was the introduction of *M. sexmaculatus* in conjunction with the flowering plant coriander. This treatment had the lowest mean value of 13.4 ± 1.6, indicating its superiority over the control group and other experimental plots. The treatment that ranked second in terms of efficacy, known as CScR, had a population size of 19.7 ± 0.9 whiteflies. It is noteworthy to emphasize that the whitefly populations reached their highest level of 23.3 ± 2 in the plots where *M. sexmaculatus* was present, and 27.8 ± 2.5 in the plots where *C. septempunctata* was present, during the third week. This trend was particularly observed in plots that lacked flowering plants. The greatest significant effects were noticed during the fifth and sixth weeks of the experiment. The populations recorded after the third application of natural enemies across all plots ranged from 10.3 ± 0.5 to 20.6 ± 1.6 nymphs and adults per plant. In the sixth week, the population numbers were recorded as follows: MScR 11.60 ± 5, MS 14.6 ± 1.5, CScR 13.5 ± 1.7, and CS with 19.8 ± 1.5. The combined interaction between *M. sexmaculatus* and the flowering plant had a pronounced influence, particularly in the fifth week, leading to a substantial reduction (Figure 4). Subsequently, there were successive reductions of 79%, 74%, 70%, and 66% in the weeks that followed. In a similar vein, the predatory capacity of *C. septempunctata* exhibited its highest degree during the sixth week, resulting in a substantial decrease of 75% in whitefly populations. This was followed by successive reductions of 70%, 64%, 59%, and 60% in the fifth, fourth, third, and second weeks of the observation period. The results highlight the consistent success of *M. sexmaculatus* and *C. septempunctata* in managing whitefly populations in the cucumber greenhouse. Specifically, the MScR treatment exhibited the best efficacy during the fifth week, while the CScR treatment showed the highest efficacy during the sixth week (Figure 4). The five plots also showed considerable variations in predator effectiveness, with some having blooming plants and others without them, as seen in Table 1. These results highlight the important role that floral resources play in the efficiency of these predators in managing whitefly populations.

### 3.3. Fecundity Performance on Floral Plant

**Figure 5:**
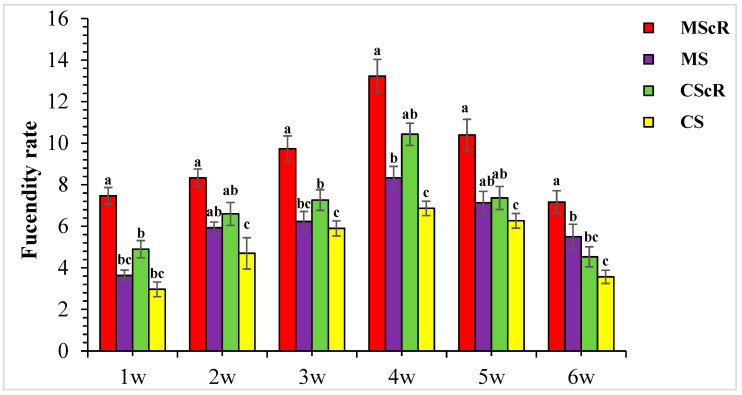
The fecundity rate of *M. sexmaculatus* and *C. septempunctata* in a cucumber greenhouse with the presence of a floral plant (*Coriandrum sativum*) and natural enemies without floral plants MScR = *M. sexmaculatus* + *C. sativum*, MS = *M. sexmaculatus*; CScR = *C. septempunctata* + *C. sativum*; CS = *C. septempunctata* at different times and W = weeks of observation. Shows the average eggs laid per plant. There was a significant effect between treatments; MS plots had significantly fewer eggs than MScR plots over time and a similar trend was observed between CS and CScR plots. ANOVAs with Tukey HSD post hoc tests were used to compare treatments. The different letters indicate statistically significant differences between treatments.

This study investigated how *C. sativum* affected the fertility rate of *M. sexmaculatus* and *C. septempunctata* while aphid pests were present. With differences observed throughout several treatments and weeks, the presence of *C. sativum* considerably affected how fertile these predators were. The data demonstrate continuous predator activity throughout the crop’s development cycle, highlighting the dynamic nature of predator–prey interactions in integrated pest-control systems. The presence of *C. sativum* had a substantial impact on the fecundity rate, with 13.2 ± 0.8 and 10.4 ± 0.6 per plant when *M. sexmaculatus* and *C. septempunctata* were present, as shown in Figure 5. During the second week of observation, the MScR plot exhibited the highest fecundity rate, with an average of 8.3 ± 0.4 eggs per plant. In contrast, the CS plot displayed the lowest fecundity rate, recording an average of 4.3 ± 0.8 eggs per plant. In a similar vein, during the third week, the MScR plot showed the highest rate of fecundity, with an average of 9.7 ± 0.6 eggs per plant. This was followed by the CScR plot, which exhibited a rate of 7.2 ± 0.5 eggs per plant. The CS plot, on the other hand, displayed the lowest rate of fecundity at 5.9 ± 0.4 eggs per plant. The fertility rate of predatory beetles showed a negative correlation with the decrease in host insect pest populations. In the sixth week of the MScR plot, the fecundity rate was recorded to be 7.1 ± 0.5 eggs per plant. On the other hand, the CScR plot exhibited the second-highest fecundity rate of 3.5 ± 0.3 eggs per plant; additionally, there were significant differences in fecundity rate by ANOVA Table 1. The results of this study indicated that the predator exhibited sustained activity throughout the whole duration of the crop’s growth cycle. The presence of *M. sexmaculatus* and *C. septempunctata* had a notable impact on the fecundity rate when *C. sativum* was present. The fecundity rates exhibited variability across different treatments and weeks, emphasizing the dynamic nature of predator–prey interactions within the framework of integrated pest-management systems.

This study aimed to investigate the effects of *M. sexmaculatus* and *C. septempunctata* on populations of whiteflies and aphids in a controlled environment of a cucumber greenhouse. The findings consistently indicated that the presence of both predators led to beneficial decreases in pest populations. Notably, the most substantial reductions were recorded when predators were paired with floral-resource plants. Furthermore, it was revealed that the precise timing of predator release played a pivotal role in attaining optimal control of aphid populations. In addition, the results suggested that these predators remained active during the entire growing period. The results of this study highlight the potential of utilizing these natural predators as a valuable component in integrated pest-management approaches for greenhouse farming.

## 4. Discussion

Agricultural environmental plans that advocate for the establishment of ecosystems abundant in flowers aim to augment biodiversity and ecosystem functions, such as pollination and pest control; nevertheless, there is limited available information about the efficacy of these practices in facilitating many ecosystem services on agricultural land. This study demonstrates the potential of utilizing floral structures as a criterion for plant species selection in cucumber greenhouses, aiming to enhance the attraction of natural enemies and, therefore, augmenting floral resources. Floral strips have emerged as a promising approach for enhancing the fitness of essential natural predators while minimizing the proliferation of detrimental insect species, ultimately resulting in enhanced pest-control services. The results of our study are consistent with prior research conducted by [31,32], which emphasizes the beneficial effects of flower strips on the overall health and effectiveness of ecologically important predators, hence enhancing pest-management services. Previous studies have demonstrated that incorporating blooming plants into outdoor farming systems can enhance the populations of advantageous insects, including hoverflies [21,33]. Growers may be hesitant to use blooming plants in commercial covered growing systems due to maintenance issues. The use of additional food sources in place of flowers to provide pollen and nectar was investigated. Hoverfly treatments efficiently reduced aphid numbers in a preventative trial using strawberry plants that were in bloom, but only when additional food sources were added with hoverflies was a substantial decrease observed [34]. Our study’s findings support the utilization of the manipulation method, including the introduction of floral resources.

Numerous methodologies have been investigated by researchers in order to augment the populations of indigenous syrphids within greenhouse environments. The successful employment of cultural strategies, such as opening greenhouse side walls, and conservation biological control techniques, such as providing aphids as a food source, has been documented [24]. The integration of these approaches inside greenhouse environments can enhance the efficacy of indigenous syrphids, which serve as valuable agents for biological pest control. In the present investigation, the intentional exposure of *M. sexmaculatus* and *C. septempunctata* to pest concentrations of *B. tabaci* and *M. persicae* was conducted, aiming to replicate the prevailing conditions observed in commercial greenhouse cropping systems. As aphids have become one of the hardest pests to control in greenhouse crops, it may be interesting to support the development and effectiveness of these aphidophagous predators [35,36]. Realistic circumstances play a crucial role in the stabilization of multi-species biocontrol agent releases, as evidenced by previous studies [37,38].

The phenomenon of predator–prey density dependency is of significant importance in the implementation of biological control initiatives. The present study elucidates the positive association between the population levels of *C. septempunctata* and *M. sexmaculatus* with the densities of aphids and whiteflies in cucumber agricultural systems. This discovery highlights the significance of taking into account both natural predators and their prey when developing efficient pest-control methods. In addition to our analysis, we also studied the impact of floral resources on the fecundity rates of *Menochilus sexmaculatus* and *Coccinella septempunctata*. The study revealed that *Coriandrum sativum* had a notable effect, as evidenced by the MScR plot where floral plants displayed the highest rate of fecundity. The results of this study align with previous research that assessed the hoverfly’s potential for biocontrol in a greenhouse test against strawberry potato aphids from both a preventive and therapeutic standpoint, as well as the results of including sugar and pollen in their diet. Contrary to prior studies, the quantity of *Sphaerophoria rueppellii* eggs increased as the number of adult hoverflies increased; this underscores the significance of floral resources in enhancing the reproductive effectiveness of natural predators, as highlighted in earlier research [25,34,39].

It is recommended to enhance the efficacy of conservation biological management by introducing a diverse array of plant species that have overlapping flowering seasons. This method enhances the accessibility of floral nutrients, thus providing advantages to beneficial insects such as syrphids. The synchronization of flowering with the peak activity time of syrphids is of utmost importance [40]. The findings of our study indicate that habitats with a high abundance of flowers have the potential to serve as substitutes for or supplements to pesticide inputs in orchards. However, it is recommended that future research investigate the effects of flowering plants in orchards with different levels of agrochemical usage [41]. The adoption of a comprehensive methodology will yield valuable perspectives on the implementation of sustainable pest-management strategies. Our study highlights the significance of ecosystems abundant in flowers and the deliberate choice of plant species based on their floral structures as means to promote biodiversity and ecosystem services, specifically pest control, within agricultural systems. The above-mentioned results hold great significance in the realm of sustainable agriculture and underscore the criticality of incorporating ecological interactions among natural predators, prey, and floral resources into pest-management approaches. Subsequent research endeavours ought to delve deeper into the reasonable implications of these findings within reliable agricultural contexts.

## 5. Conclusions

In conclusion, our study underscores the considerable potential of landscapes abundant in flowers and the deliberate selection of plant species based on their floral structures to augment biodiversity, ecosystem services, and pest management within agricultural systems. This strategy is consistent with conservation biological control strategies, which provide ecological advantages. The findings of our research indicate that the augmentation of *Menochilus sexmaculatus* and *Coccinella septempunctata* populations in the presence of *Coriandrum sativum* plants has the dual effect of increasing reproductive capacity and improving the effectiveness of biocontrol agents, while simultaneously diminishing populations of target pests. The findings mentioned above underscore the need for considering ecological interactions when implementing pest-management strategies, and propose practical implications for promoting sustainable agricultural practices.

## Figures and Tables

**Table 1:** The ANOVA results indicate that there is a significant difference between a floral plot and a non-floral plot by the mean population of *Myzus persicae*, *Bemisia tabaci*, and the fecundity rate of *Menochilus sexmaculatus* and *Coccinella septempunctata* in the greenhouse experiment.

	3 Days	1st Week	2nd Week	3rd Week	4th Week	5th Week	6th Week
*Myzus persicae*							
*F*	13.84	5.82	6.82	155.34	6.01	6.03	489.6
*df*	4	4	4	4	4	4	4
*p*	0.0059	0.0423	0.0311	0.0231	0.0398	0.0396	0.0482
*Bemisia tabaci*							
*F*	6.95	8.47	14.63	12.72	8.86	9.08	7.70
*df*	4	4	4	4	4	4	4
*p*	0.0299	0.0196	0.0051	0.0073	0.0177	0.0167	0.0241
Fecundity rate							
*F*		10.42	10.50	6.76	25.22	9.99	62.86
*df*		2	2	2	2	2	2
*p*		0.0233	0.0230	0.0380	0.0040	0.0251	0.0376

## Data Availability

All data are available in all figures and tables of the manuscript.
